# Non-invasive cardiac output measurement with electrical velocimetry in patients undergoing liver transplantation: comparison of an invasive method with pulmonary thermodilution

**DOI:** 10.1186/s12871-018-0600-y

**Published:** 2018-10-02

**Authors:** De-Jie Wang, I-Shan Lee, An-Hsun Chou, Chun-Yu Chen, Pei-Chi Ting, Yun-Hui Teng, Jr-Rung Lin, Hsin-I Tsai

**Affiliations:** 1 Department of Anesthesiology, Chang Gung Memorial Hospital, Linkou Medical Center, Chang Gung University, No. 5, Fusing St, Guishan District, Taoyuan City, 33305 Taiwan; 2 Graduate Institute of Clinical Medical Sciences, College of Medicine, Chang Gung University, Taoyuan, 333 Taiwan; 3 Clinical Informatics and Medical Statistics Research Center and Graduate Institute of Clinical Medicine, Chang Gung University, Taoyuan, Taiwan

**Keywords:** Cardiac output, Pulmonary thermodilution, Electrical velocimetry, Liver transplantation

## Abstract

**Background:**

The goal of this study was to evaluate the accuracy and interchangeability between continuous cardiac output (CO) measured by electrical velocimetry (CO_Ev_) and continuous cardiac output obtained using the pulmonary thermodilution method (CO_PAC_) during living donor liver transplantation (LDLT).

**Method:**

Twenty-three patients were enrolled in this prospective observational study. CO was recorded by both two methods and compared at nine specific time points. The data were analyzed using correlation coefficients, Bland-Altman analysis for the percentage errors, and the concordance rate for trend analysis using a four-quadrant plot.

**Results:**

In total, 207 paired datasets were recorded during LDLT. CO data were in the range of 2.8–12.7 L/min measured by PAC and 3.4–14.9 L/min derived from the EV machine. The correction coefficient between CO_PAC_ and CO_Ev_ was 0.415 with *p* < 0.01. The 95% limitation agreement was − 5.9 to 3.4 L/min and the percentage error was 60%. The concordance rate was 56.5%.

**Conclusions:**

The Aesculon™ monitor is not yet interchangeable with continuous thermodilution CO monitoring during LDLT.

**Trial registration:**

The study was approved by the Institutional Review Board of Chang Gung Medical Foundation in Taiwan (registration number: 201600264B0).

## Background

During the living donor liver transplantation (LDLT), the hemodynamic status changes dramatically. Sudden blood loss, clamping of the great vessels and reperfusion syndrome may cause hemodynamic instability. Moreover, the clinical features of cirrhotic patients include high cardiac output (CO), low systolic vascular resistance (SVR) and tachycardia [1]. Thus, perioperative management becomes extraordinarily challenging for anesthesiologists. Standard intraoperative monitoring includes CO monitoring, which allows the anesthesiologist to make prompt and accurate decisions when needed. The gold standard of CO measurement during liver transplantation is the thermodilution technique using a pulmonary artery catheter (PAC) [2]. However, complications have been reported regarding the placement of the PAC, such as pneumothorax, air embolus, arrhythmia, right bundle branch block, catheter knotting, thrombosis [3], right ventricular rupture [4] or pulmonary artery rupture [5]. Whether there is an additional benefit to decision making provided by a PAC over standard care in elderly, high-risk surgical patients requiring intensive care [6–9] is questionable. That said, the importance of intraoperative CO monitoring has led to the development of non-invasive or less-invasive cardiac output monitors, which appear promising as replacements for the thermodilution technique in the intensive care unit; however, none of them can provide adequate precision and accuracy during LDLT [10–12].

Electrical velocimetry (EV), a type of non-invasive cardiac output monitoring based on thoracic electrical impedance, during cardiac ejection only uses four standard electrocardiographic electrodes to measure CO. Two electrodes are placed at the base of the neck on the left side and the other two on the left inferior part of the thorax at the level of the xiphoid process. EV is measured based on the changes in thoracic electrical impedance as the ohmic equivalent of the mean aortic blood flow acceleration. The stroke volume (SV (mL)) and CO (L/min) can be derived by the following equations:

$$ SV= VEPT\times v\times LVET $$

$$ CO= SV\times HR/1000 $$

In these equations, VEPT (mL) is the volume of electrically participating tissue calculated from the body mass and body height, ν (/s) is mean aortic blood flow velocity during left ventricular ejection, LVET (s) is the left ventricular ejection time and HR (beat/min) is the heart rate. The Aesculon™ bioimpedance electrical cardiometry monitor (Osypka Medical GmbH, Berlin, Germany) is based on the EV formula.

Studies have been conducted to assess the accuracy of CO measurement using EV compared to thermodilution, Fick equation and transthoracic doppler echocardiography [13–16]. In post-cardiac surgical patients, the accuracy and interchangeability of electrical velocimetry with the thermodilution method has been demonstrated [17–20]. Rajput RS et al. applied the EV device during cardiac surgery and showed that the percentage error ranged from 22 to 32% [21].

However, limited data are available to evaluate the accuracy and precision of EV during LDLT. The aim of this study is to assess whether non-invasive EV can replace the continuous thermodilution technique (by PAC) during LDLT.

## Materials and methods

This study was a prospective observational study. The study was approved by the Institutional Review Board of Chang Gung Medical Foundation in Taiwan (registration number: 201600264B0). Informed consent forms were obtained from all the participants of the study. Twenty-three patients undergoing LDLT in Chang-Gung Memorial Hospital between July 2016 and March 2017 were enrolled in this study. The exclusion criteria were preoperative atrial fibrillation, significant valvular pathology, intracardiac shunt, severe pulmonary hypertension and refusal to provide consent.

General anesthesia was started with propofol, 1 to 2 mg/kg; fentanyl, 1 to 2 mg/kg; and cisatracurium, 0.2 mg/kg or rocuronium 1.2 mg/kg. For the cardiac output measurement, the PAC was placed through the right internal jugular vein and a triple lumen central venous catheter was inserted through the left internal jugular vein. The tip position of the PAC was confirmed by the waveforms of pulmonary artery pressure. The PAC was connected to a Vigilance II Monitor (Edwards Lifesciences, USA). The continuous thermodilution machine measures CO every 30 s. CO measured by EV was obtained by the Aesculon™ monitor. After the PAC was inserted, four surface electrodes were applied to the patient according to the Aesculon™ protocol. As the triple lumen catheter was fixed on the left side of neck, two electrodes were applied on the left side of the cheek and neck. The other two electrodes were applied on the lower thorax. Only signals with adequate data quality were included in the analysis.

After the sensors were attached to the skin, the data were checked to ensure proper functioning of the sensors. The clock in the operation room, on the Aesculon™ and on the continuous thermodilution machine were synchronized. CO_Ev_ and CO_PAC_ were documented at nine time points: (1) immediately after PAC placement and calibration, T1; (2) 60–90 min after skin incision, T2; (3) 120–150 min after skin incision, T3; (4) immediately after removing the liver, T4; (5) 15 min after removing the liver, T5; (6) 30 min after removing the liver, T6; (7) immediately after releasing the inferior vena cava clamp, T7; (8) 15 min after releasing the inferior vena cava clamp, T8; (9) 30 min after releasing the inferior vena cava clamp, T9. The data were classified into three phases: dissection phase (T1-T3), anhepatic phase (T4-T6), and reperfusion phase (T7-T9).

During the LDLT, the partial clamp on the inferior vena cava (piggyback technique) was used in our hospital. Piggyback technique was proved to be a safer approach to venous outflow tract reconstruction from the hemodynamic point of view [22].

### Statistical analysis

Statistical analysis was performed using SPSS 22.0 (SPSS Inc., Chicago, IL, US) and R version 3.0.4 (Vienna, Austria). The correlation coefficient and Bland-Altman analysis were used to assess the agreement between two methods [23]. A correlation coefficient between 0.9 and 1.0 indicates a strong correlation; a correlation coefficient < 0.5 indicates a weak correlation. Bias, limitation of agreement and percentage error were calculated. The percentage error (1.96* standard deviation/average of CO_PAC_ and CO_Ev_) was calculated according to Critchley and Critchley [24]. The clinically acceptable percentage error is less than 30%. The trending ability was assessed using a four-quadrant plot [25]. The central exclusion zone of the four-quadrant plot was ±1.0 L/min for small changes in CO [26]. The concordance rate was defined as the percentage of the total number of plots in the first and third quadrants of the four-quadrant plot. The concordance rate was considered good and clinically acceptable if the rate exceeded 92%. Pearson’s correlation was also performed to assess the association between the two methods. Statistical significance was set at *p* < 0.05.

## Results

Twenty-five patients who underwent planned LDLT between July 2016 and March 2017 were initially enrolled in this study. However, two patients were subsequently excluded, leaving data from 23 patients for analysis. One patient was excluded because of a poor electrical signal, so the CO could not be calculated by the EV machine. The operation was canceled for the other patient due to high pulmonary arterial pressure. The baseline demographic data are shown in Table 1.

**Table 1:** Patient characteristics of liver transplantation recipients

Characteristic	Descriptive statistics
Age (years)	56 ± 7(41–68)
Gender	
Male	18
Female	5
Body mass index(kg/m2)	24.5 ± 3.0(20.4–31.9)
Ascites amount (ml)	2265 ± 3744(0–12,400)
Indication for LDLT	
HBV related HCC	11
HCV related Cirrhosis	4
HBV with acute liver failure	3
Alcoholic related cirrhosis	2
Drug	1
HBV related cirrhosis	2
MELD SCORE	18 ± 11(6–41)
< 10	5
10–19	10
20–29	4
> =30	4

Data are described as mean ± standard deviation (range) or number

*LDLT* Living donor liver transplantation, *HBV* Hepatitis B virus, *HCC* hepatocellular carcinoma, *HCV* Hepatitis C virus, *MELD* Model for End-Stage liver disease

A total of 207 paired data sets were recorded. CO data were in the range of 2.8–12.7 L/min measured by PAC and 3.4–14.9 L/min derived from the EV machine, as revealed in Table 2. The highest intragroup correction coefficient was 0.646 at the time point immediately after removing the inferior vena cava partial clamp. The overall mean CO_PAC_ was 7.1 ± 2.2 L/min and the mean CO_Ev_ was 8.4 ± 2.2 L/min. The average CO_Ev_ was slightly higher than the average CO_PAC_. The correction coefficient between CO_PAC_ and CO_Ev_ was 0.415 with *p* < 0.01. The correction coefficients were 0.553 in the dissection phase, 0.276 in the anhepatic phase and 0.376 in the reperfusion phase (Table 3).

**Table 2:** Correction coefficient, bias and 95% limitation of agreement of all measurements and time point

Time	Intragroup correction coefficient	Bias (L/MIN)	Limits of agreement(L/MIN)	CO_PAC_ (L/MIN)	CO_Ev_ (L/MIN)	SVR by PAC dyne*cm^−5^
T1	***0.592	−0.78	− 4.38 to 2.82	6.70 ± 2.02	7.49 ± 2.05	908 ± 460
T2	***0.524	−0.65	− 4.92 to 3.62	6.88 ± 2.18	7.54 ± 2.28	897 ± 553
T3	***0.555	−1.03	−4.87 to 2.81	7.00 ± 2.24	8.03 ± 1.85	862 ± 410
T4	***0.466	−1.20	− 5.75 to 3.35	6.99 ± 2.34	8.19 ± 2.13	794 ± 662
T5	0.334	−1.59	−6.67 to 3.49	6.83 ± 2.14	8.41 ± 2.37	829 ± 344
T6	0.012	−2.29	−7.74 to 3.16	6.61 ± 2.14	8.90 ± 1.80	896 ± 750
T7	***0.646	−2.43	−6.25 to 1.39	6.81 ± 2.29	9.23 ± 2.33	863 ± 364
T8	0.276	−0.62	−5.27 to 4.03	8.06 ± 1.96	8.68 ± 1.99	616 ± 230
T9	0.304	−0.72	−6.35 to 4.91	8.40 ± 1.99	9.13 ± 2.77	665 ± 233

Abbreviations: *CO*_*PAC*_ cardiac output derived from continuous transpulmonary thermodilution method, *CO*_*Ev*_ cardiac output derived from Electrical velocimetry method

Data are shown as mean ± standard deviation

*** the *P*-value < 0.05

**Table 3:** Correction coefficient, bias and percentage error between CO_PAC_ and CO_Ev_ measurement in each surgical phase

Phase	correction coefficient	Bias (L/MIN)	Limits of agreement(L/MIN)	Percentage error (%)
Total	***0.415	−1.26 ± 2.39	− 5.94 to 3.42	60.0%
Dissection	***0.553	−0.82 ± 1.97	− 4.68 to 3.04	53.1%
Anhepatic	***0.276	−1.69 ± 2.57	−6.73 to 3.34	65.7%
Reperfusion	***0.376	−1.26 ± 2.53	− 6.22 to 3.70	59.1%

Data are shown as mean ± standard deviation

*** the *P*-value < 0.05

**Fig. 1:**
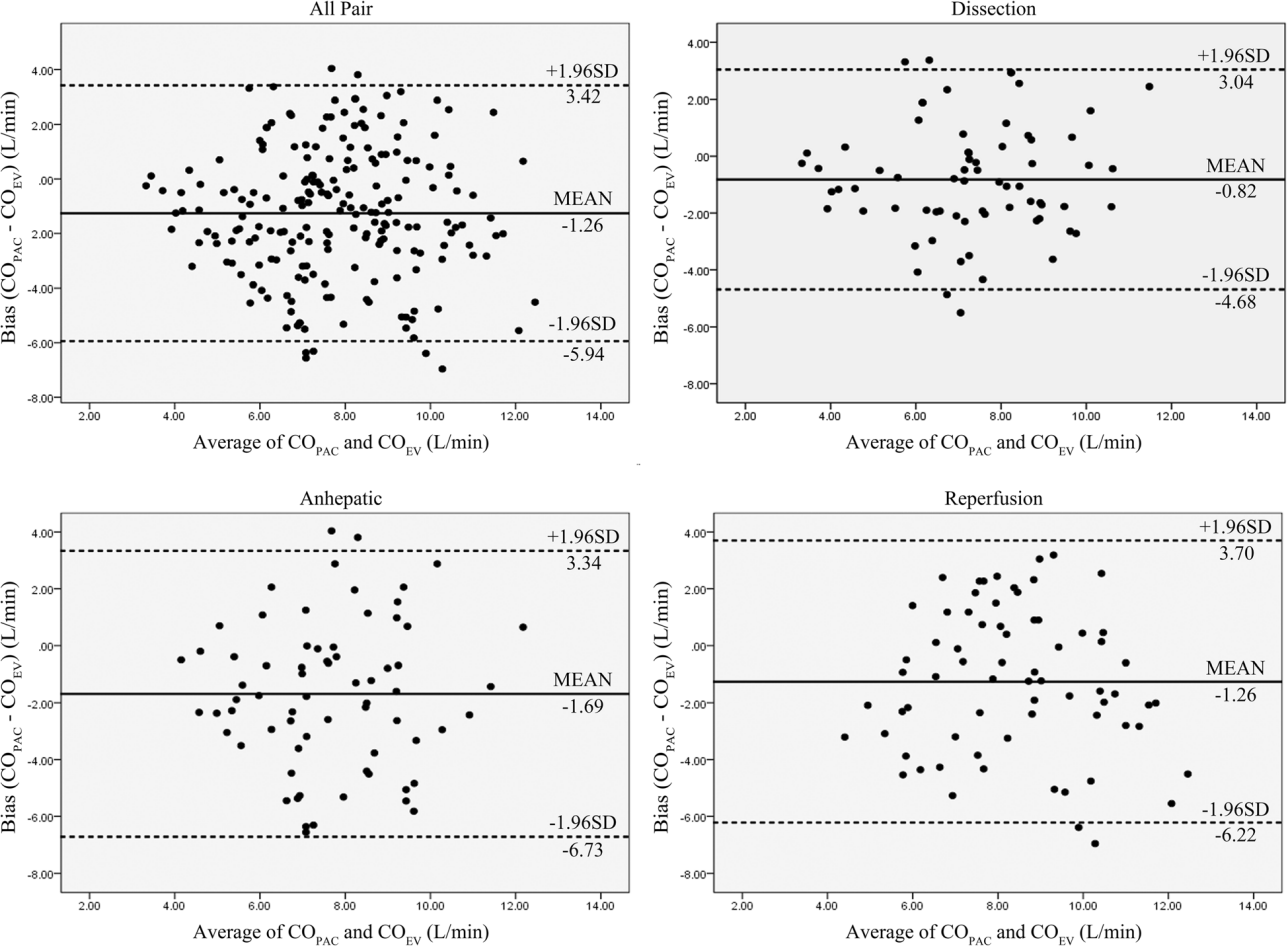
Bland-Altman plot for CO_Ev_ and CO_PAC_. Bias and limits of agreement (±1.96SD) are shown in the plot. Abbreviations: CO_PAC_ cardiac output derived from continuous transpulmonary thermodilution method; CO_Ev_ cardiac output derived from Electrical velocimetry method; SD standard deviation

**Fig. 2:**
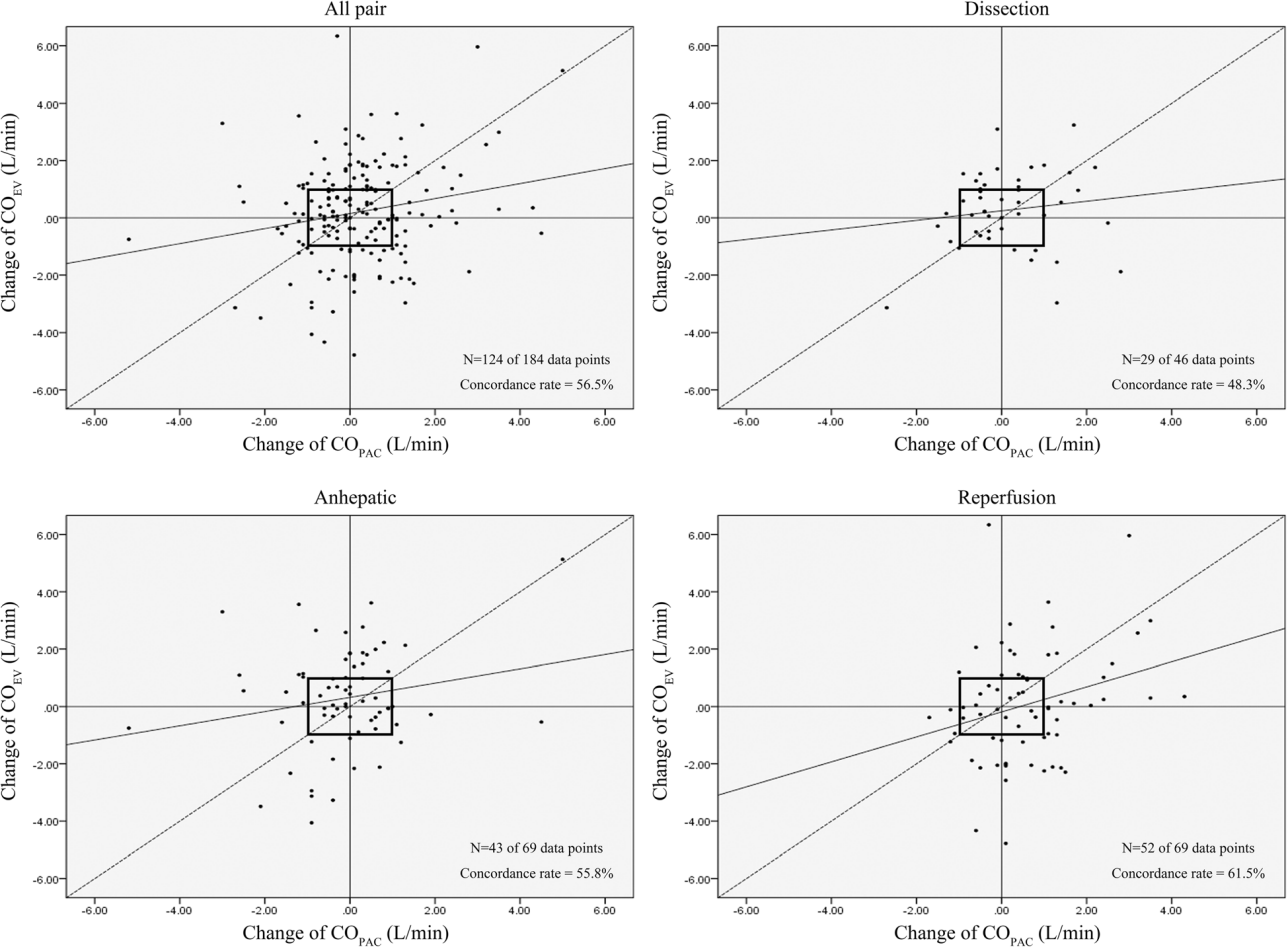
Four-quadrant plot for comparing changes in CO_Ev_ and CO_PAC_. Data points within the ±1.0 L/min exclusion zone (box in central) are considered statistical noise and excluded. The dot line represents the line x = y. The N means the number of plots out of the exclusion zone. Abbreviations: CO_PAC_ cardiac output derived from continuous transpulmonary thermodilution method; CO_Ev_ cardiac output derived from Electrical velocimetry method

The Bland-Altman analysis with a mean bias between CO_PAC_ and CO_Ev_ was − 1.26 L/min and the 95% limitation agreement was − 5.9 to 3.4 L/min (Fig. 1). The overall percentage error was 60%, which failed to meet the criterion of interchangeability (< 30%). Figure 2 shows the Bland-Altman plot in three phases. The percentage errors were 53% in the dissection phase, 65.7% in the anhepatic phase and 59.1% in the reperfusion phase.

The SVR data calculated from the Vigilance II Monitor were also collected. The overall mean of SVR was 815 ± 383 dyne*cm^− 5^ (mean ± SD). The mean of SVR dropped from 863 dyne*cm^− 5^ to 615 dyne*cm^− 5^ at T8 or 15 min after liver reperfusion. There was a negative correction between the bias of CO_PAC_ from CO_Ev_ and SVR (*r* = − 0.317 *p* < 0.01). The highest intragroup correction coefficient was − 0.67 at T6, or 30 min after removing the liver.

Four-quadrant plots were drawn to evaluate the trending ability, as shown in Fig. 2. The central exclusion zone was set at ±1 L/min. The CO_PAC_ changes and CO_Ev_ changes were compared. In total, 184 paired data sets were placed on the four-quadrant plots. There were 60 sets within the central exclusion zone. After excluding the central zone data, the concordance rate between the two methods was 56.5%. The concordance rates were 48.3% in the dissection phase, 55.8% in the anhepatic phase and 61.5% in the reperfusion phase.

## Discussion

In our study, the continuous thermodilution method was utilized to measure CO instead of the gold standard using the intermittent thermodilution technique, in which the accuracy can be affected by the timing of the injection within the respiratory cycle, the change of pulmonary artery blood temperature, the injectate, the speed of the injection and the placement of the catheter [13]. Under hemodynamically stable condition, a good correction, accuracy and precision between continuous and intermittent cardiac output measurement during has been shown in the literature [27–29]. The limitation agreement between the continuous thermodilution method and the intermittent thermodilution technique is clinically acceptable. Bottiger BW et al. [30] has demonstrated significant correlation between intermittent and continuous CO measurements (*r* = 0.87, *p* < 0.0001), accompanied with a bias of 0.240 L/min during orthotopic liver transplantation. They also revealed that the changes in the pulmonary artery blood temperature would influence the CO measurements more by intermittent thermodilution than by continuous thermodilution during reperfusion. The continuous CO monitor was therefore used to determine the reliability of EV-based CO measurements. We found that CO_Ev_ showed limited accuracy when compared to continuous thermodilution CO assessment.

This study demonstrates that the Aesculon™ system using the EV formula is not interchangeable with the established automatic thermodilution method using a PAC in patients undergoing LDLT. The percentage error was 60% and was not clinically acceptable. In our study, CO_Ev_ was generally higher than CO_PAC_. The mean bias between CO_PAC_ and CO_Ev_ was − 1.26 L/min. Several factors could have contributed to such poor interchangeability between the Aesculon™ system and thermodilution using a PAC during LDLT. First, in the equivalent of CO_Ev_, the VEPT (mL) is related to the patient’s body weight [31]. VEPT could be miscalculated in the presence of a large volume of ascites. As ascites is one of the clinical manifestations in patients with liver cirrhosis requiring LDLT, the presence of ascites may cause an overestimation of the patient’s body weight, leading to an overestimation of CO by the Aesculon™ monitor. In our study, the amount of ascites varied greatly among the patients, ranging from 0 ml to 12,000 ml. In the extreme situation, the ascites could make up to 20% of the body weight overestimation, which in turn may give a greater value CO_Ev_ than CO_PAC_. Secondly, the massive ascites could displace the upward the diaphragm upwards, causing geometric changes that could alter the conductivity of the thorax and thus affect the accuracy of the EV method. Thirdly, surgical manipulation of the upper abdomen could have an impact on the CO_Ev_. Surgical interventions to the upper abdomen could cause a shift in the bioimpedance cardiac output index readings by > 1 L/min/m^2^, and the direction of the shift was unpredictable [32]. The use of surgical retractor in addition to abdominal wall compression may additionally contribute to a change in conductivity. A poor correlation between those two methods was noted in this study. The correction coefficient between CO_PAC_ and CO_Ev_ was 0.415 with *p* < 0.01. The subgroup analysis showed that correlation coefficients were 0.553 in the dissection phase, 0.276 in the anhepatic phase and 0.376 in the reperfusion phase. The relatively stable hemodynamics during dissection may be the reason why this phase was shown to have a better correlation coefficient. Manipulation of the great vessels during the anhepatic phase could cause a decrease in the preload, and renal vein congestion may cause hemodynamic instability [33]. Reperfusion syndrome may also lead to low blood pressure during the reperfusion phase [34, 35]. The reason why hemodynamic stability affected the correction coefficient was due to the measurement interval of those two techniques. The CO_Ev_ measurement was based on a change in conductivity and measured CO in seconds. However, the continuous CO_PAC_ measurement needs several minutes [36]. Even though the readings on the CO_PAC_ monitor refreshed CO every 30 s, the CO displayed was the average of CO over the preceding 3–6 min [30]. When the CO changes, the CO_PAC_ machine needed more time to determine the real CO. With hemodynamic disturbances, CO_Ev_ could be assumed to be closer to real-time CO than CO_PAC_, which could be minutes behind. The best intragroup correction coefficient was achieved immediately after the inferior vena cava partial clamp was released, possibly as a result of the relatively hemodynamic stability and minimal interruption of the electrical signal during vessel anastomosis. It takes minutes to impact the hemodynamic after off-clamp. Due to the relatively slow response of the continuous thermodilution measurement, the correction coefficients were much worse during hemodynamic instability.

The concordance rate was 56.5% when the central exclusion zone was ±1 L/min, much lower than the clinically acceptable concordance rate of 92%. This may also be attributed by the lag in CO measurement using the continuous thermodilution technique.

This study has several limitations. First, the EV used four electrodes to detect the signal and calculate the CO. However, electro-coagulation and cutting could interfere with these signals during surgery. With every interruption, the EV machine required approximately at least 30 s to reacquire the conduction signal. The artefacts overlaying the recorded signal may in fact cause an over- or underestimation of CO, in particularly during the dissection phase. In extreme situations, the EV machine could only acquire one data point per hour due to interference from electro-coagulation. EV interference could also be due to mechanical compression of the thoracic electrodes by the surgeon. Second, the small sample size warrants further studies with a larger population size. Third, the trending ability of the EV monitor was only partially surveyed. During LDLT, fluid challenge, inotropic agent usage and great vessel clamping could cause hemodynamic changes.

## Conclusions

In conclusion, the Aesculon™ monitor exhibited limited accuracy, precision and trending ability when compared to continuous thermodilution CO monitoring during LDLT. The Aesculon™ system is not yet interchangeable with continuous thermodilution cardiac output monitoring during LDLT.

## Abbreviations

CO: Cardiac output
CO_Ev_: Cardiac output measured by electrical velocimetry
CO_PAC_: Cardiac output measured by the pulmonary thermodilution
PAC: Pulmonary artery catheter
LDLT: Living donor liver transplantation
EV: Electrical velocimetry
RBCs: Red blood cells
SV: Stroke volume
VEPT: Volume of electrically participating tissue
ν: Mean aortic blood flow velocity during left ventricular ejection
LVET: Left ventricular ejection time
HR: Heart rate
SD: Standard deviation
